# Abnormal rib count in scoliosis surgery: impact on the reporting of spinal fusion levels

**DOI:** 10.1007/s11832-014-0623-y

**Published:** 2014-11-05

**Authors:** Hillard T. Spencer, Meryl E. Gold, M. Timothy Hresko

**Affiliations:** Department of Orthopaedic Surgery, Boston Children’s Hospital, Harvard Medical School, 300 Longwood Avenue, Hunnewell 2, Boston, MA 02115 USA

**Keywords:** Scoliosis, Abnormal rib count, Vertebral numbering variation, Spinal fusion

## Abstract

**Purpose:**

Variation in rib numbering has been noted in adolescent idiopathic scoliosis (AIS), but its effect on the reporting of fusion levels has not been studied. We hypothesized that vertebral numbering variations can lead to differing documentation of fusion levels.

**Methods:**

We examined the radiographs of 161 surgical AIS patients and 179 control patients without scoliosis. For AIS patients, the operative report of fusion levels was compared to conventional vertebral labeling from the first thoracic level and proceeding caudal. We defined normal counts as 12 thoracic (rib-bearing) and five lumbar (non-rib-bearing) vertebrae. We compared our counts with data from 181 anatomic specimens.

**Results:**

Among AIS patients, 22 (14 %) had an abnormal number of ribs and 29 (18 %) had either abnormal rib or lumbar count. In 12/29 (41 %) patients, the operative report differed from conventional labeling by one level, versus 3/132 (2 %) patients with normal numbering (*p* < 0.001). However, there were no cases seen of wrong fusion levels based on curve pattern. Among controls, 11 % had abnormal rib count (*p* = 0.41) compared to the rate in AIS. Anatomic specimen data did not differ in abnormal rib count (*p* = 1.0) or thoracolumbar pattern (*p* = 0.59).

**Conclusions:**

The rate of numerical variations in the thoracolumbar vertebrae of AIS patients is equivalent to that in the general population. When variations in rib count are present, differences in numbering levels can occur. In the treatment of scoliosis, no wrong fusion levels were noted. However, for both scoliosis patients and the general population, we suggest adherence to conventional labeling to enhance clarity.

## Introduction

Variations in rib and vertebral numbering occur both in the general population and in other groups [1, 2]. Such variations may include alterations in the total number of vertebral levels or may simply affect the number of levels identified with a particular segment, such as the thoracic or lumbar spine [3]. The presence of an abnormal rib count has been noted in some patients with adolescent idiopathic scoliosis (AIS), but its effect on surgical treatment, or on documentation, has not been described [4]. Ambiguity in the labeling of spinal levels may arise when variation is present. Other studies have noted that wrong-level spinal surgery is the most frequent wrong-site procedure in orthopedics, and that anatomic variation is a risk factor [5–9]. However, in scoliosis, the fusion levels are based on global curve characteristics, including magnitude, stiffness, and sagittal profile, and no study has examined whether numerical variation has any effect on scoliosis surgery.

The enumeration of ribs is, perhaps, the simplest method for the radiographic examination of variation in spinal segmentation through the thoracolumbar region [10]. The historical teaching has been that 2–8 % of individuals in the general population will have just 11 sets of ribs [1, 11]. In addition, transitional lumbosacral vertebrae have been shown in other studies to be very common, but as they are outside the region of most idiopathic scoliosis fusion cases, they are not individually classified in this present study [12–15]. We hypothesized that vertebral numbering variations in the thoracolumbar region can lead to ambiguous documentation of fusion levels in AIS.

## Materials and methods

This study was performed at a tertiary children’s hospital with Institutional Review Board approval. A retrospective review of medical records was performed on scoliosis patients within a previous prospective study at our institution and control patients that were identified from radiology department records. All surgical cases were performed by experienced pediatric spinal surgeons and all operative reports were dictated by the attending surgeon. We defined normal counts as 12 thoracic (rib-bearing) and five lumbar (non-rib-bearing) vertebrae. Prior to data collection, statistical power analysis showed that a sample size of 160 patients was required in each group to detect a difference of 10 % at a significance level of 0.05 with 80 % power. For the purpose of this study, we classified levels according to the conventional schema utilized by Pilbeam [16], after Schultz, designating thoracic vertebrae based on an articulation with a rib and lumbar vertebrae as fully segmented presacral vertebrae not articulating with a rib. This method simplifies phenotypic classification and avoids attempts to differentiate whether the underlying developmental source of variation is homeotic (shift of a regional boundary) or meristic (subtraction of a segment). For consistency with the selected classification schema, fully segmented mobile vertebrae at the lumbosacral junction were considered lumbar, and those that articulate or fuse by the transverse process on one or both sides with sacrum are counted as a half lumbar and sacral, respectively [16].

We examined 164 consecutive patients enrolled in a separate prospective study of AIS at our institution between 2003 and 2005 who underwent spinal fusion. Any patient with magnetic resonance imaging (MRI) findings of intraspinal pathology suggesting an underlying etiology for the scoliosis was excluded. Spinal fusion levels were selected by each treating surgeon in accordance with widely taught principles, and cases underwent peer review in surgical conference with preoperative and postoperative radiographs. Three patients were excluded for age below 10 years, leaving 161 patients in the scoliosis group. For AIS patients, the report of levels fused in surgery was compared to a conventional vertebral count beginning with the first thoracic level (T1) and proceeding caudal. There were no neurologic complications in the surgical group. For the control group, we identified 212 consecutive patients between the ages of 10 and 21 years who underwent chest radiography in our emergency room in the summer of 2006. To be included, each chest radiograph had to show a vertebral level above and below the rib cage and have sufficient resolution to visualize the ribs. We excluded 25 patients who were noted to have some degree of scoliosis or a subsequent diagnosis of scoliosis in later medical records. In addition, seven patients with radiographs that did not visualize the entire rib cage and one patient with a vertical expandable prosthetic titanium rib device (VEPTR) device for thoracic insufficiency syndrome were excluded, leaving 179 patients in the control group. A subset of AIS radiographs was reviewed individually by two different surgeons to calculate a kappa statistic for inter-rater agreement. Fisher’s exact test was utilized to compare the number of cases classified as normal or abnormal number of ribs between groups. Finally, to generalize comparisons of both thoracic and lumbar counts to a population outside of our medical center, all data were compared to counts from 181 anatomic specimens, previously published elsewhere (Table 1) [16]. Significance was set at *p* = 0.05. All *p*-values were two-sided.

**Table 1:** Summary of thoracic and lumbar count data on anatomic specimens, *n* = 181 (collated from Table 1 in Pilbeam [16])

	Lumbar count					Total
Thoracic count	4	4.5	5	5.5	6	
11	0	0	1	0	1	2
11.5	0	2	1	0	0	3
12	3	2	144	3	5	157
12.5	0	3	4	0	0	7
13	8	1	3	0	0	12
Total	11	8	153	3	6	181

## Results

### Rib count

The characteristics of the study patients and controls are listed in Table 2, and the rib and lumbar counts are listed in Table 3. There were significantly more female patients in the scoliosis group than the control group (*p* < 0.001), consistent with the widely known demographics of surgical AIS, but the rate of abnormal rib count did not differ by sex (*p* = 0.61). Out of 161 AIS patients meeting the inclusion criteria, 22 patients (14 %) had an abnormal rib count, and only 132 (82 %) had a normal thoracolumbar pattern (12 rib pairs and five lumbar segments). There was no consistent relationship between an unpaired rib and curve convexity. In the control population, 179 met the inclusion criteria and 19 (11 %) of those patients had a rib numbering variation, which was not statistically different from the rate in AIS patients (*p* = 0.41). Incidentally, 70 of the control patients were also discovered to have imaging of the abdomen or lumbar spine and only 54 of them (77 %) had a normal thoracolumbar pattern, but this subsample size was underpowered for analysis, albeit with a non-significant *p*-value compared to AIS patients. Published data on 181 anatomic specimens showed 157 (86.7 %) with 12 rib sets and 144 (79.6 %) with the normal thoracolumbar pattern, and did not differ significantly from AIS patient data (*p* = 1.0 for rib count and *p* = 0.59 for normal thoracolumbar pattern, respectively) [16]. There were no patients with 12.5 rib pairs noted in our AIS group, though that pattern appeared in the controls and anatomic specimen data. Cervical ribs were found in one AIS patient (bilaterally) and in none of the control patients.

**Table 2:** Summary characteristics of the study population

Comparison of control and scoliosis patients	Control (*n* = 179)	Scoliosis (*n* = 161)	*p*-Value
Mean age (years)	15	14.9	0.71
Sex			
F	81	127	
M	98	34	<0.001
Rib count abnormal, *n* (%)	19 (10.6)	22 (13.7)	0.41

Comparison of rib count by sex	Rib count abnormal, *n* (%)		*p*-Value
	Yes	No	
Sex			
F	181 (87.0)	27 (13.0)	0.61
M	118 (89.4)	14 (10.6)

**Table 3:** Rib and lumbar count data for scoliosis and control patients

Scoliosis patients (*n* = 161)						
Rib count	Lumbar count					Total
	4	4.5	5	5.5	6	
11	0	0	10	0	5	15
11.5	0	1	0	2	0	3
12	2	2	132	1	2	139
12.5	0	0	0	0	0	0
13	1	0	3	0	0	4
Total	3	3	145	3	7	161

Control patients (*n* = 179) with chest radiographs	
Rib count	*n*
11	7
11.5	4
12	160
12.5	8
13	0
Total	179

Subset of control patients with additional lumbar imaging (*n* = 70)						
Rib count	Lumbar count					Total
	4	4.5	5	5.5	6	
11	0	0	2	0	0	2
11.5	0	2	0	1	0	3
12	4	0	54	0	2	60
12.5	0	0	0	0	0	0
13	1	0	4	0	0	5
Total	5	2	60	1	2	70

### Surgical treatment

**Fig. 1:**
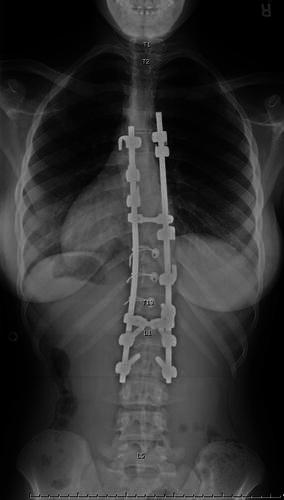
The operative report for this patient with 13 ribs stated that T5–L2 was instrumented, but the conventional count would be T6–L2

**Fig. 2:**
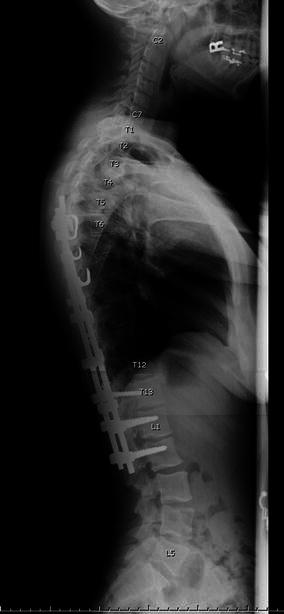
Lateral radiograph of the same patient as in Fig. 1 confirms the extra thoracic level is present

We reviewed the postoperative radiographs for all patients in the AIS group and counted the levels included in the fusion using the first thoracic vertebra as the reference (“conventional numbering”). We then reviewed the attending surgeon’s operative report for every patient. Among the 29 patients with abnormal vertebral counts, there were 12 operative reports (41 %) in which the surgeon’s labeling of the levels of fusion differed by one level compared to conventional numbering (example in Figs. 1 and 2), corresponding to the use of a numbering schema from the thoracolumbar junction. Among the 132 patients with normal vertebral counts, there were three such operative reports (2 % of cases), one of which was a patient with hypoplastic T12 ribs. The difference in this frequency between the normal and abnormal groups of AIS patients was highly statistically significant (*p* < 0.001). In the group with abnormal vertebral counts, the discrepancy was significantly associated with variation in the number of thoracic vertebrae (*p* = 0.006), occurring in nine patients with 11 rib sets and three patients with 13 rib sets, but was not associated with a variation in the number of lumbar vertebrae (*p* = 0.236). There were only three operative reports (10 % of 29 patients) in which the surgeon had specifically described the numbering convention being utilized in the presence of a variation in spine anatomy. However, there were no cases determined to have undergone a wrong-level spinal surgery or any incorrect choice of fusion levels in the global treatment of the curve, or to have suffered any complication attributable to the numbering convention selected.

### Radiology reports

We reviewed the official radiology reports of all radiographs for the 29 AIS patients with variations in rib or lumbar vertebral counts. For 15 patients, the variation was not seen in any of the radiology reports before or after surgery; for five patients, it was noted in at least one radiology report preoperatively; and for nine patients, it was only mentioned after surgery. For two patients with preoperative radiology recognition, the operative report still differed by one level compared to a conventional count from T1 (Table 4). Reviewing the official radiology reports for the 19 patients in the control group who had an abnormal rib count, we did not find any instances in which the radiologist had commented on the difference in rib number (*p* < 0.001 compared to the AIS group). The interobserver kappa was 1.0 for rib count only (*p* < 0.0001, 100 % agreement), but this dropped to 0.64 (*p* = 0.0010, 95 % agreement) when including the lumbar count, with disagreement occurring over the classification of a lumbosacral transitional vertebra.

**Table 4:** Operative reports and radiology reports in 29 adolescent idiopathic scoliosis (AIS) patients who had abnormal rib or lumbar count

Patient number	Number of rib pairs	Number of lumbar vertebrae	Instrumented levels according to operative report	Levels instrumented, conventional count from T1	Comparison of operative report vs. count from T1	Was a variation described in operative report?	Was variation ever noted by radiology?	When did radiology report the variation?
217	13	5	T11–L3	T12–L3	Different			
230	13	5	T5−L2	T6–L2	Different		Yes	Postop
233	11.5	5.5	T10–L2	T10–L2				
250	12	6	T3–L3	T3–L3				
255	12	5.5	T4–L1	T4–L1				
259	12	6	L1–L4	L1–L4			Yes	Preop
267	11	5	T4–L2	T3–L2	Different			
270	12	4	T1–T6	T1–T6			Yes	Postop
271	11	5	T2–T10	T2–T10				
273	11	5	T3–T12	T2–T11	Different			
277	11	6	T11–L2	T11–L2		Surgeon noted		
284	12	4	T4–T11	T4–T11			Yes	Preop
286	11	5	T3–L2	T3–L2				
289	12	4.5	T4–L3	T4–L3			Yes	Preop
290	11	5	T3–T11	T3–T11				
291	12	4.5	T10–L2	T10–L2			Yes	Postop
292	11	6	T5–L3	T4–L3	Different		Yes	Preop
294	11	5	T3–L3	T2–L3	Different			
299	13	4	T3–L2	T4–L2	Different		Yes	Postop
300	11.5	5.5	T12–L3	T12–L3				
303	11	5	T3–L3	T2–L3	Different		Yes	Postop
305	11	5	T4–L1	T3–L1	Different			
314	11	6	T1–L1	T1–L1		Surgeon noted “L1 (T12)”	Yes	Postop
324	11	6	T11–L2	T11–L3	Different		Yes	Postop
334	11	6	T6–L2	T6–L3	Different		Yes	Preop
343	11	5	T5–T12	T4–T11	Different			
345	11	5	T2–L3	T2–L3				
366	13	5	T4–T12	T4–T12	(Cervical ribs)	Surgeon noted T12 small	Yes	Postop
369	11.5	4.5	T5–T11	T5–T11			Yes	Postop

## Discussion

Variations in the number of ribs or of lumbar vertebrae occur both in the general population and in patients with idiopathic scoliosis. However, this is the first study to show that these variations are associated with differences in the reporting of fusion levels during AIS surgery, but that this difference in the numbering convention did not lead to any wrong fusion levels. In the 12 operative reports that differed from a conventional numbering schema, all patients had an abnormal rib count (either 11 or 13 pairs). The abnormal rib count was usually not described in radiology reports, shifting responsibility to the treating surgeon to recognize these variations, as has been suggested elsewhere [4]. In our study, there were no cases seen of incorrect selection of fusion levels, likely because fusion levels are selected based on curve characteristics rather than on the numerical designation of a spinal segment. However, our study also shows that these numerical variations are equally common in the general population without scoliosis.

There are numerous studies of wrong-level spine surgery, but none have specifically focused on scoliosis like the present study. A report from the American Board of Orthopaedic Surgery (ABOS) showed wrong-level spinal surgery to be the most common wrong-site surgery in orthopedics [5]. Other studies have shown that anatomic variation can lead to the incorrect localization. One group reported that a wrong-level thoracic discectomy occurred in a patient who had both cervical ribs and absent T12 ribs, a variation that was not recognized until after surgery [7]. Another case report documented a discectomy performed at the wrong level in a patient with cauda equina syndrome who had a lumbarized S1 vertebra, unrecognized preoperatively [8]. Furthermore, patients with variations in the number of lumbar vertebrae may have non-classical dermatomyotomal supply patterns, creating a challenge for the accurate clinical diagnosis of radicular symptoms [17, 18]. Other studies have identified unconventional spine anatomy and counting differently compared to radiology to be among several factors that contributed to wrong-level operations [6, 9, 19]. Therefore, to provide clarity, the surgeon should verify the numbering convention used to localize pathology before surgery for any patient with a variation in vertebral count.

We compared our data with published anatomic specimen data from the anthropology literature to confirm that these findings were not limited to our patient population [16]. Indeed, another report of 1,239 anatomic specimens showed only 1,031 (83.2 %) to have exactly 12 thoracic and five lumbar vertebral levels [20]. Although a prior study suggested only moderate agreement (kappa of 0.53) between observers using standard radiographs to detect transitional lumbosacral vertebrae, our study showed that the rib count had excellent (kappa of 1.0) interobserver reliability on plain radiographs, and for the lumbar count moderate (kappa of 0.64) agreement, which was due to differing interpretation of a transitional vertebra [21]. The apparently low kappa even in the presence of a high percentage of agreement may be due to the paradoxical property of the kappa statistic in the presence of low prevalence findings [22, 23]. In addition, the very fact that disagreement in numbering can arise in patients with transitional vertebrae as seen in this study underscores the importance of preoperative recognition and attendant surgical planning.

There may be several reasons why the radiologists’ reports did not routinely include a mention of the numbering variation. First, it is possible that, in some instances, it was not noted. Second, because our spinal radiographs were obtained in patients with scoliosis, the pathology is generalized rather than focal, and it is not necessary to establish a precise frame of reference while generally describing the spinal curve. Third, the radiologist may feel that such details are superfluous, as he or she encounters such variation frequently in the course of work, or may be pressed for time and dictate from a template which leaves out such information. However, the need to comment on such findings would become paramount when identifying a focal pathology, such as a tissue lesion, fracture, or herniated disk. In the literature reviewed above, the absence of a clear comment on numerical variation was felt to partially contribute to the occurrence of wrong-level surgery in many cases. In addition, in a fee-for-service healthcare system, billing codes for spinal fusion are based on the number of levels included, and failure to identify numerical variations may lead to coding inaccuracies unless the specific rib counting method is stated in the operative report.

Certain limitations are present in this study. Although no cases of wrong-level spine surgery were found when examining the choice of AIS fusion levels based on curve characteristics, it may be difficult to extrapolate these findings to other spinal surgeries that involve only a specific spinal segment. Nonetheless, our finding that the labeling of fusion levels differed from conventional numbering frequently in patients with abnormal thoracolumbar anatomy highlights the importance of communication and explicit documentation of anatomic variation preoperatively. Because a large sample of full-length spinal radiographs was not readily available for normal patients, we used sequential chest radiographs performed in the emergency room at our institution as a control population for the rib count. To address the absence of lumbar radiographs for most of these control patients, we performed comparison of lumbar variation with published anatomic specimen data, a decision that gave this study the added benefit of generalization to a population outside of our institution. Therefore, despite its limitations, this study conclusively demonstrates that vertebral numbering variation is similarly common in patients both with and without spinal deformity, and that such variation may impact the surgeon’s choice of numbering system for the operative spinal levels.

In conclusion, accurate recognition and description of numerical variations in spinal segmental anatomy is mandatory prior to operative treatment of spine pathology. The rib count is a highly reliable method of identifying many of these variations in the thoracolumbar region. For patients with scoliosis, such recognition will allow unambiguous description of the levels included in the fusion construct. We suggest adherence to conventional labeling from the first thoracic level and proceeding caudal. For both scoliosis patients and the general population, numbering variations should be noted preoperatively to enhance clarity.
